# From data to diagnosis: how machine learning is revolutionizing biomarker discovery in idiopathic inflammatory myopathies

**DOI:** 10.1093/bib/bbad514

**Published:** 2024-01-17

**Authors:** Emily McLeish, Nataliya Slater, Frank L Mastaglia, Merrilee Needham, Jerome D Coudert

**Affiliations:** Murdoch University, Centre for Molecular Medicine and Innovative Therapeutics, Murdoch, Western Australia (WA), Australia; Murdoch University, Centre for Molecular Medicine and Innovative Therapeutics, Murdoch, Western Australia (WA), Australia; Perron Institute for Neurological and Translational Science, Nedlands, WA, Australia; Murdoch University, Centre for Molecular Medicine and Innovative Therapeutics, Murdoch, Western Australia (WA), Australia; Perron Institute for Neurological and Translational Science, Nedlands, WA, Australia; University of Notre Dame Australia, School of Medicine, Fremantle, WA, Australia; Fiona Stanley Hospital, Department of Neurology, Murdoch, WA, Australia; Murdoch University, Centre for Molecular Medicine and Innovative Therapeutics, Murdoch, Western Australia, WA, Australia; Perron Institute for Neurological and Translational Science, Nedlands, WA, Australia; University of Notre Dame Australia, School of Medicine, Fremantle, WA, Australia

**Keywords:** idiopathic inflammatory myopathies, machine learning, biomarkers, myositis-specific autoantibodies

## Abstract

Idiopathic inflammatory myopathies (IIMs) are a heterogeneous group of muscle disorders including adult and juvenile dermatomyositis, polymyositis, immune-mediated necrotising myopathy and sporadic inclusion body myositis, all of which present with variable symptoms and disease progression. The identification of effective biomarkers for IIMs has been challenging due to the heterogeneity between IIMs and within IIM subgroups, but recent advances in machine learning (ML) techniques have shown promises in identifying novel biomarkers. This paper reviews recent studies on potential biomarkers for IIM and evaluates their clinical utility. We also explore how data analytic tools and ML algorithms have been used to identify biomarkers, highlighting their potential to advance our understanding and diagnosis of IIM and improve patient outcomes. Overall, ML techniques have great potential to revolutionize biomarker discovery in IIMs and lead to more effective diagnosis and treatment.

## INTRODUCTION

Idiopathic inflammatory myopathies (IIMs) encompass a diverse group of disorders, including adult dermatomyositis (ADM), juvenile dermatomyositis (JDM), anti-synthetase syndrome (ASS), overlap myositis (OM), polymyositis (PM), immune-mediated necrotising myopathy (IMNM) interchangeably referred to as necrotising autoimmune myopathy and sporadic inclusion body myositis [1]. Biomarkers are ‘a defined characteristic that is measured as an indicator of normal biological processes, pathogenic processes or responses to an exposure or intervention’ [2]. They have emerged as powerful tools for diagnosis, predicting disease prognosis and identifying therapeutic targets. For example, lymphocytes-expressing Bcl-2 and CCR4 are indicative of anti-HMGCR+ IMNM [3], and DM skeletal muscle biopsies have upregulated interferon (IFN)-stimulated gene signatures, indicating a role for type 1 IFNs in DM pathogenesis [4, 5]. These findings have led to promising mechanism-based treatments, such as tofacitinib or ruxolitinib (JAK/STAT inhibitors), which have been shown to reduce serum IFN-I levels and improve skin lesions and muscle weakness in DM patients [6, 7].

However, the heterogeneity in symptoms and disease progression within IIM subgroups often poses additional challenges to identifying effective biomarkers. Thus, a successful biomarker would not only accurately distinguish IIM from other conditions that can present with similar symptoms, such as muscular dystrophies or metabolic myopathies but also differentiate one IIM from another [8]. In addition, the capacity to identify patients across the spectrum of disease severities or to stratify rapidly progressing patients would be invaluable for disease management. Currently, a thorough evaluation that includes a combination of clinical, laboratory, radiological and pathological assessments is necessary to establish an accurate diagnosis.

This paper reviews recent studies on potential biomarkers for IIM and assesses their clinical utility. We also explore data analytic tools and machine learning (ML) algorithms that have proven valuable for biomarker discovery, highlighting their potential to advance our understanding of IIM and improve patient outcomes.

### ML approaches for biomarker discovery

ML algorithms have revolutionized this field of biomedicine. Inflammatory myopathies have been investigated using various ML techniques, such as clustering algorithms, principal component analysis (PCA) and deep neural networks. These models learn complex relationships between variables, handle missing or noisy data, and assist in making real-time predictions [9]. In addition to diagnosing diseases, ML algorithms provide valuable insights into therapeutic outcomes in various diseases, allowing clinicians to tailor treatment plans based on a patient's predicted response to therapy. It is important to note, however, that further research is still needed to validate their accuracy and determine their clinical utility. Nonetheless, the potential for ML to revolutionize biomarker discovery and therapeutic outcomes in various diseases including IIM is becoming increasingly evident.

**Figure 1:**
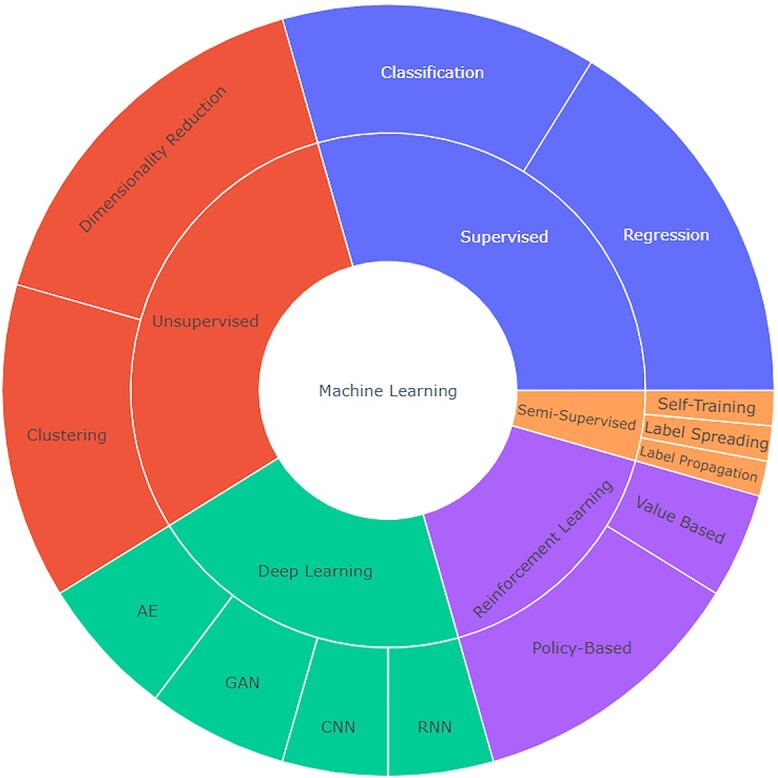
Interactive starburst plot showing machine learning categories, subsets, and algorithms. The interactive starburst plot displays the main categories of machine learning, including supervised and unsupervised learning, and their corresponding subsets and algorithms. Users can explore the plot to gain insights into the various machine learning techniques and their applications. This is an interactive plot: follow link: https://chart-studio.plotly.com/∼Emilymc/3.embed GAN: generative adversarial networks, RNN: recurrent neural networks, CNN: convolutional neural networks, AE: autoencoders, BIRCH: Balanced Iterative Reducing and Clustering using Hierarchies, OPICS: Ordering Points To Identify the Clustering Structure, SARSA: State-Action-Reward-State-Action.

ML algorithms can be classified into five main categories (Figure 1): supervised, unsupervised, semi-supervised, reinforcement learning and deep learning [9, 10].

In supervised learning, the algorithm is trained on labelled data to learn mapping from inputs to outputs. This approach is best applied for classification or regression tasks [9]. Examples include decision trees and support vector machines (SVMs).

In unsupervised learning, the algorithm is trained on unlabelled data to find patterns or relationships within the data [10]. This method is used for clustering or dimensionality reduction tasks. Examples include k-means clustering and PCA.

Semi-supervised learning involves training an algorithm from both labelled and unlabelled data. In this approach, the algorithm is provided with some labelled data to learn from, and then it translates this knowledge to make predictions on the unlabelled data.

In reinforcement learning, the algorithm learns to make decisions based on feedback from its environment [10]. This is often used in game playing or robotics. Examples of these algorithms include Q-learning and deep reinforcement learning networks.

Deep learning involves training artificial neural networks to recognize patterns in data. These neural networks are made up of layers of interconnected nodes that process and transform input data to produce an output [11]. These networks often possess multiple layers, allowing them to learn complex representations of the input data. Deep learning has been particularly successful in applications such as image and speech recognition, natural language processing and autonomous driving [11].

### ML modelling versus statistical modelling

**Figure 2:**
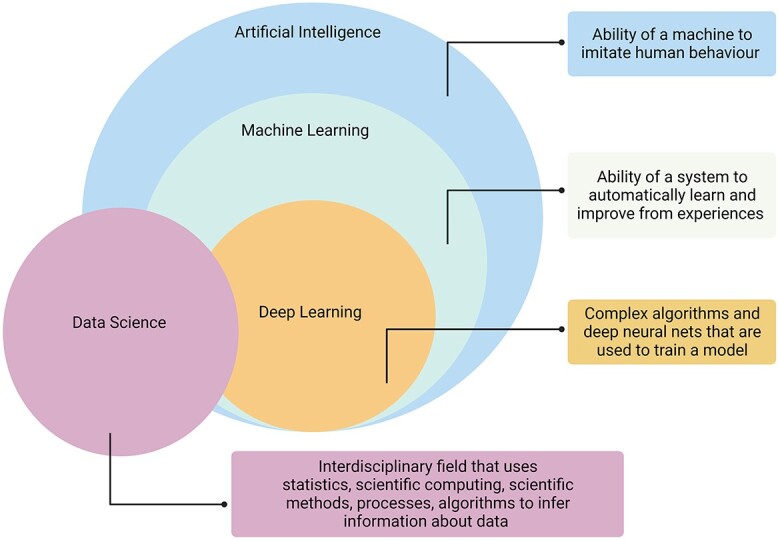
The relationship between artificial intelligence, machine learning, deep learning and data science. The diagram highlights how these fields build on each other to provide advanced solutions for data-driven problems. Figure created with Biorender.com

ML and statistical modelling (SM) are two popular approaches for analyzing medical and scientific data (Figure 2). Although ML and SM are related fields, they are not synonymous. Statistics is a branch of mathematics that deals with collecting, analyzing and interpreting data. ML, on the other hand, constitutes a branch of artificial intelligence dedicated to devising algorithms and models that enable computers to acquire knowledge from data and generate predictions [12]. Although ML has a strong mathematical foundation, incorporating statistical techniques like inference, hypothesis testing and regression analysis, it also extends beyond traditional methods with techniques like neural networks, deep learning and natural language processing to address complex problems.

In some cases, ML and SM overlap, as seen with logistic regression (LR) analysis. LR is conventionally considered a statistical model that determines the odds ratio (OR) based on binary outcomes, where the dependent variable has two possible categorical values [13]. For example, the binary outcome of not having or having a disease can be represented as 0 and 1, respectively. However, LR can also be viewed as a supervised ML model since it utilizes a training dataset to learn the relationship between predictor variables and the binary outcome. Once trained, the LR model can make predictions on new data by estimating the probability of the binary outcome [14].

One of the key differences between ML and SM is the focus of each field. Statistics is primarily concerned with comparing and summarizing data, while ML is focused on building predictive models [12, 15]. Furthermore, statistics typically deal with relatively small and carefully defined datasets, while ML mostly involves working with large and complex datasets.

ML models are powerful tools for exploratory research because they identify complex patterns in large and high-dimensional datasets; they can also handle missing data. They make no prior assumptions about the data and are flexible as they can be trained on new data input that becomes available. SM is a valuable approach, particularly when the underlying mechanisms of the data are known, and when the research question is confirmatory [12]. Both methods have their advantages and disadvantages, and researchers must consider their research aims and the nature of their data before deciding which approach is the most appropriate.

### ML models for diagnosis of IIM and subgroups of IIM

Accurate diagnosis of a type of IIM is often challenging, as many muscle conditions possess overlapping clinical features and laboratory findings [8]. Recent ML models have been applied to multiple different patient datasets, including clinical, histopathological and imaging data and have provided new opportunities for improving the accuracy and speed of IIM diagnosis.

Earlier diagnostic criteria for IIM such as the Bohan and Peter criteria predominantly focused on distinguishing between DM and PM as many other myopathies such as IBM and IMNM had not yet been separated from PM [16]. These initial classifications heavily relied on clinical presentation and histology, both of which required a high level of medical expertise for interpretation (Table 1: *Polymyositis and dermatomyositis diagnostic criteria*). However, later adaptions of the Bohan and Peter criteria utilized ‘computer-assisted analysis’ although it was unclear what methods specifically this refers to in this study [17]. More recent criteria for IIM combine the clinical, histological and serology (predominantly myositis-specific or myositis-associated antibodies (MSA); see below). However, in many of these earlier publications, validation of the specificity and sensitivity for these criteria were either not performed at the time or have been conducted in later studies [18–34] (Table 1).

**Table 1:** Specificity, sensitivity and characteristics of various diagnostic and classification criteria for IIM adapted from Lundberg *et al.* [34]

Criteria	Symptom duration	Age	Muscle weakness	Muscle pain	Muscle biopsy	EMG	Muscle enzymes	Extramuscular features	MSA	Sensitivity and specificity
**Polymyositis and dermatomyositis diagnostic criteria**										
Medsger *et al*. [19]		X	X	X	X	X	X			Not validated
DeVere and Bradley [20]			X	X	X	X	X	X		Not validated
Bohan and Peter [16]			X		X	X	X	X		94.3%; 29.4% Validated by [18]
Dalakas [21]			X		X	X	X	X		88.6%; 47.1% Validated by [18]
Tanimoto *et al*. [22]			X	X	X	X	X	X	X	88.6%; 29.4% Validated by [18]
Targoff *et al*. [23]			X		X	X	X	X	X	97.1%; 29.4% Validated by [18]
Dalakas and Hohlfeld [24]			X		X	X	X	X		77.1%; 99.9% Validated by [18]
Hoogendijk *et al*. [25]		X	X		X	X	X	X	X	71.4%; 82.4% Validated by [18]
Oddis *et al*. [26]			X		X	X	X	X		93%; 93%
**IBM-specific diagnostic criteria**										
Griggs criteria [27]	X	X	X		X	X	X			Sensitivity: 11%–100% Specificity: 73%–100% Validated by [29] [38]
2000 European Neuromuscular Centre (ENMC) criteria [28]		X	X		X	X	X	X		Sensitivity: 46%–65% Specificity: 98%–100% Validated by [38]
2010 MRC Centre for Neuromuscul. Dis. [30]	X	X	X		X	X	X			Sensitivity: 11%–73% Specificity: 98%–100% Validated by [38]
2013 European Neuromuscular Centre (ENMC) criteria [32]	X	X	X		X	X	X			Sensitivity: 15%–84% Specificity: 98%–100% Validated by [38]
Lloyd et al. [38]			X		X					90%; 96%
^a^Criteria based on high-performing features from other criteria.										
**IMNM-specific diagnostic criteria**										
Triplett et al. [37]		X	X		X	X	X		X	AUC ROC 97.1%
224^th^ ENMC International Workshop (32)		X	X		X	X	X	X	X	93%; 88% Validated by [1]
**Overlap myositis specific criteria**										
Troyanov *et al.* [39]		X	X					X	X	Sensitivity 87%
^a^Modified Bohan & Peter clinic-serological classifications										
**All IIM diagnostic criteria**										
2017 EULAR/ACR [1]		X	X		X		X	X	X	93%; 88%; Reviewed by [33] Sensitivity 80.9–99.6%; Reviewed by [36]
Mariampillai *et al.* [40]		X	X					X	X	77%; 92%
Eng *et al.* [35]			X				X	X	X	AUROCs between 78% and 97% and AUPRCs between 55% and 96% for individual MSA

A recent diagnostic data-derived criteria for IIM is the 2017 European League Against Rheumatism/American College of Rheumatology (EULAR/ACR) classification criteria [1] (Table 1: *All IIM diagnostic criteria*). This criterion includes 12 clinical features including age, gender, the pattern of muscle weakness, skin manifestations, laboratory features such as elevated serum creatine kinase (CK) concentrations, presence of autoantibodies and histopathological features including the pattern of inflammation, perifascicular atrophy and vacuoles [1]. Each feature is scored, and the total value translates into diagnosis classification as either ‘definite’, ‘probable’ or ‘possible’ JDM, DM, ADM, IBM, or PM. The sensitivity and specificity of the 2017 (EULAR/ACR) classification were evaluated at 93% and 88%, respectively, and were greater when a muscle biopsy had been performed. Nonetheless, the criteria still performed well without a biopsy [1].

**Figure 3:**
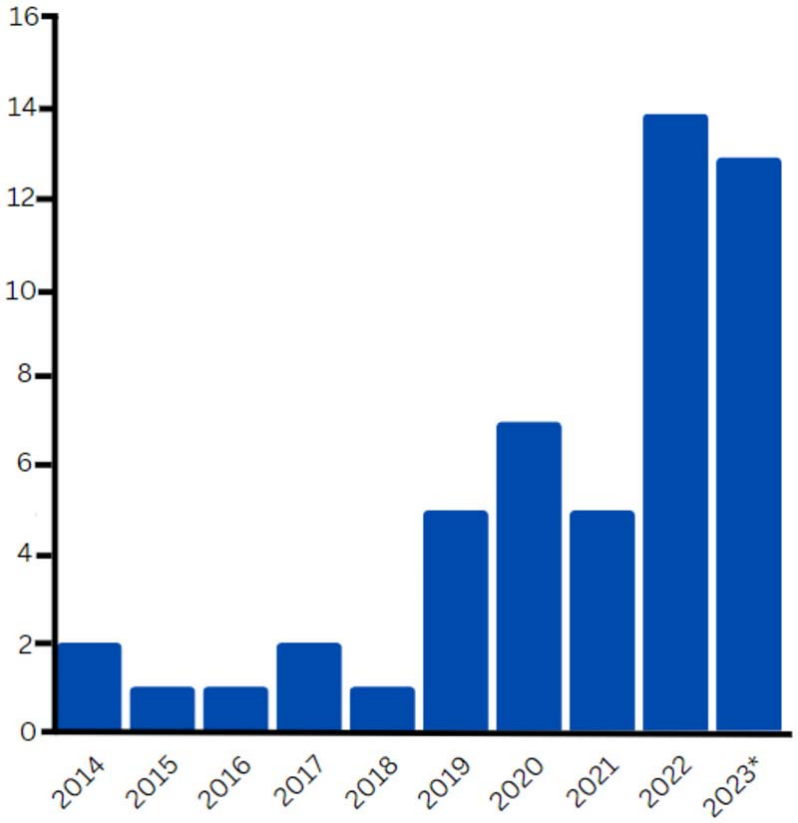
Number of publications using ML algorithms in IIM research. Bar graph showing the number of publications investigating the use of ML in the field of IIM between 2014 and between January and October 2023^*^. The data presented herein have been derived from PubMed and are reflective of publications available as of 4 October 2023. These publications were identified using specific search criteria, employing the terms ‘Inflammatory myopathies and Machine Learning’. Figure created with Biorender.com

Despite significant progress in the diagnostic criteria of IIM, it is not exempt from limitations. One notable example is its failure to account for the disease heterogeneity and distinguish between IMNM and PM, while also being unable to assess myositis-specific autoantibodies other than beyond anti-Jo-1. As a result, there are still challenges to accurately classify and subtype patients [35, 36]. This has highlighted the need for identifying novel biomarkers that can aid in the diagnosis, classification and prognostication of IIM, and ultimately improve patient outcomes. Over the last two decades, significant advances in computational capabilities have resulted in the development of more powerful ML models that are increasingly being applied in the medical field. The number of yearly publications that used ML for diagnostic and subclassification of IIMs increased by nearly 10 times between 2014 and May 2023 (Figure 3). ML has the potential to effectively tackle the heterogeneity of IIMs, offering a promising avenue to enhance the accuracy of predicting disease progression and outcome.

In 2020, a study by Triplett *et al.* [37], (Table 1: *IMNM specific diagnostic criteria*) described a novel criterion for diagnosing IMNM using ML regression analysis. In a cohort of 119 IMNM patients and 938 with other types of myopathy, a multivariate regression analysis of 20 variables identified eight predictors, including statin exposure, increased CK levels (>1000 U/l), and muscle weakness in the deltoid gluteus maximus and finger extensors; while finger flexor and ankle dorsiflexor were unaffected, and lastly electrical myotonia could also accurately distinguish IMNM from other myopathies (97% area under the curve for receiver operating characteristic [AUC ROC]). The authors determined that electrical myotonia was much more significant in IMNM than other forms of myopathy and could help improve the diagnosis of IMNM, particularly in cases where the disease has a chronic and indolent course, and where patients test negative for autoantibodies against hydroxy-3-methylglutaryl-coenzyme-A reductase (HMGCR) or signal recognition particle (SRP54) [37].

Since 1987, 24 diagnostic criteria for IBM have been proposed by IBM experts (Table 1:* IBM-specific diagnostic criteria*). Although some of these criteria showed high specificity (97% or higher), their sensitivities varied widely. In response to this, Lloyd and coworkers [38] developed a new diagnostic criterion for IBM based only on the most effective features from the previous criteria and constructed using a range of classification ML algorithms. It includes a combination of three main parameters: weakness of finger flexors or quadriceps, endomysial inflammation, and invasion of non-necrotic muscle fibres or rimmed vacuoles. This new criterion was tested on 371 patients and reported a sensitivity of 90% and a specificity of 96%.

OM is a term used to describe patients with an inflammatory myopathy that occurs together with other connective tissue disorders (CTDs) such as systemic sclerosis (SSc, scleroderma), systemic lupus erythematosus (SLE), rheumatoid arthritis (RA), Sjogren’s syndrome (SS), or mixed connective tissue diseases (MCTDs) [39]. Alternatively, some authors consider a definition of OM if certain MSA are present even without clinical features of CTD. The diagnostic classification of OM was reviewed by Troyanov and colleagues who developed a new classification system, placing overlap features at the core, and compared it with the original Bohan and Peter classification [39]. They found that the modified classification that includes overlap antibodies has led to an increased frequency of OM diagnosis than the original classification. The modified classification also showed better sensitivity for identifying OM patients. The authors identified different types of OM-related antibodies that can be used as biomarkers for different disease courses and treatment responses. They proposed that the new classification has diagnostic, prognostic and therapeutic value and should replace the original classification (Table 1: *Overlap myositis specific criteria*).

In a study by Eng *et al.* [35], IIM patients from a previous Rituximab trial were stratified into five groups using similarity network fusion (SNF). SNF is a powerful ML approach for combining multiple types of biological and clinical data and is designed to uncover hidden relationships and clusters within patients by leveraging similarity information between data points. The five patient group assignments were then predicted using a sparse multinomial regressor. The outcomes, denoted by area under the receiver operating characteristic (AUROCs) ranging from 78% to 97%, and area under the precision-recall curve (AUPRCs) spanning from 55% to 96%, indicate the feasibility of stratifying IIM groups based on the presence of MSA. The presence of anti-Mi-2 and anti-synthetase autoantibodies (more commonly referred to as anti-histidyl tRNA synthetase antibodies) was observed in adult DM. Conversely, anti-NXP2 autoantibodies were associated with juvenile DM. Furthermore, within the PM subgroup, notable observations included a reduction in IgM levels and the presence of anti-SRP autoantibodies. While these findings might align more closely with the features of INMN, it is imperative to consider that the study participants were enrolled prior to the introduction of the INMN classification. Consequently, their inclusion in the PM subgroup, despite the characteristic findings, is rooted in the context of the study's pre-existing classification criteria [35].

In another study, Mariampillai and colleagues stratified IIM patients using unsupervised multiple correspondence analysis and hierarchical clustering [40]. This approach successfully categorized the patients into four well-defined groups DM, IBM, IMNM and ASS; the patients who had previously been diagnosed with PM fell into the IMNM or ASS groups, suggesting that PM was no longer considered a separate diagnosis. Additionally, the algorithm showed that once again, MSA and myositis-associated antibodies (MAAs) were crucial for IIM classification, emphasizing the utility of autoantibody detection for accurate diagnosis [40]. Further study will determine whether serological testing could replace the need for muscle biopsies. Both of these studies have highlighted the potential of unsupervised ML algorithms for identifying clinically and biologically homogeneous patient groups and underscored the unique contribution of ML for identifying biomarkers for IIM.

### Clinical utility of autoantibodies in IIM

Myositis-specific antibodies being detected in approximately 60–70% of affected IIM patients have emerged as pivotal biomarkers and offer a unique lens to dissect the heterogeneity within the IIM spectrum. Anti-Jo-1 autoantibodies are detected in ASS, while anti-Mi-2, which represent the most extensively studied MSAs, is specific for DM [41]. Other examples of MSAs that are strongly associated with IMNM include antibodies to the SRP and to 3-hydroxy-3-methylglutaryl CoA reductase (HMGCR) [42, 43]. Anti-cytosolic 5′-nucleotidase 1 A (cN1A) antibodies have been found in autoimmune diseases such as SS and SLE; however, in the context of IIMs, they are restricted to IBM [44, 45].

The clinical utility of MSAs extends beyond their ability to differentiate subgroups of IIMs. They have demonstrated an association with distinct clinical attributes, thereby aiding in prognosis prediction and treatment planning. For instance, the presence of certain MSAs, like anti-TIF1-γ, anti-NXP2 and anti-HMGCR, has long been linked to an elevated risk of malignancy in IIM patients (described more in detail below) [46–49]. Moreover, anti-MDA5 antibodies, which are often found in amyopathic DM, and are a risk factor for rapidly progressive interstitial lung disease (ILD), particularly among Eastern-Asian populations [41]. Furthermore, the presence of anti-HMGCR IMNM, particularly when statin-associated, are often associated with a good response to treatment [42] alternatively, approximately 30% of IMNM patients with anti-SRP antibodies are often refractory to steroid treatments [43].

Embracing the capability of unsupervised hierarchical clustering analysis, Allenbach and colleagues [50] explored the phenotypic landscape of anti-MDA5 antibody positive patients and found that patients could be stratified into three different subgroups. In the initial subset, patients faced a rapidly progressing ILD which also corresponded to an elevated mortality rate. The second cluster predominantly displayed dermatological and rheumatological symptoms, offering a more favourable prognosis. Lastly, the third group exhibited severe skin vasculopathy, were mostly male, and had an intermediate prognosis in comparison to the other two patient groups [50].

The presence of anti-cN1A antibodies has been proposed as a potential biomarker for IBM but its diagnostic and prognostic significance remains uncertain. Sensitivity and specificity of anti-cN1A detection for IBM diagnosis have shown wide variability, ranging from 32.8% to 88.6% and 80% to 100%, respectively [45]. A meta-analysis by Mavroudis and colleagues explored its diagnostic utility using Bayesian models [51]. Bayesian models are a statistical approach that integrates prior knowledge, sourced from previous studies or expert assumptions about the data. These models are updated based on new data or information, generating posterior probabilities. This adaptability makes them valuable in decision-making processes and modelling scenarios with uncertain or limited data. While not strictly considered ML, Bayesian models are used in data analysis and diagnostics [52]. Contrary to other studies, they found that anti-cN1A antibodies could not effectively discriminate IBM from other conditions like PM/DM and MND. However, the variability in testing methodologies used across studies has introduced potential bias, given the lack of standardized protocols [51]. Furthermore, the prognostic value of anti-cN1A antibodies in IBM has produced inconsistent conclusions. With several studies noting limited prognostic value [53–55], while another study reported that seropositive patients showed increased mortality risk, less proximal upper limb weakness at disease onset, and an increased cytochrome c oxidase (COX)-deficient muscle fibres [56].

The use of ML algorithms to identify unique MSA profiles associated with distinct clinical features has greatly improved IIM stratification. However, it is important to acknowledge that there are various methods used for antibody detection, with each method having varying degree of specificity and sensitivity [57]. As discussed in the instance with anti-cN1A antibodies in IBM, discrepancies in methodologies can lead to contradictory results regarding the autoantibodies’ clinical utility. This illustrates the crucial point that computational methods’ reliability for biomarker discovery are limited by the accuracy of the detection methods. As such, careful consideration and validation of detection methodologies are imperative for accurate and meaningful results.

### Immunophenotyping as a tool for identifying biomarkers in inflammatory myopathies

**Figure 4:**
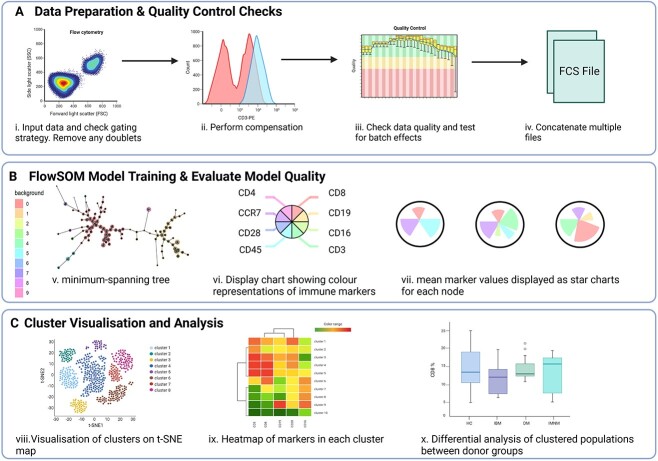
Pipeline for the main steps in the FlowSOM analysis. (**A**) Data Preparation and quality control checks (i) The fcs-files are read, (ii) compensated, (iii) QC checked and (iv) concatenated. (**B**) FlowSOM model training and evaluation of model quality. (v) The model is trained and visualization is shown as a minimum-spanning tree, which is composed of multiple inter-connecting nodes. (vi) Each node comprises a start chart of different colours representing an immune marker. (vii) Example of a start chart with mean immune marker values. (**C**) Analysis of FlowSOM model using other visualization tools such as (viii) clustering analysis via t-SNE map, (ix) heatmaps or (x) differential analysis which can be used to infer biological conclusions about the data. Figure created with Biorender.com

Immunophenotyping has become an essential tool for uncovering novel biomarkers for inflammatory myopathies. This entails a systematic exploration of the subsets, activation state and differentiation pattern of immune cell across various biological samples such as peripheral blood, muscles and other affected tissues. This approach can also involve deciphering the cytokines and chemokines that these cells produce. While certain studies have utilized ML algorithms to analyze extensive immunophenotyping data produced by techniques like flow cytometry and mass cytometry, there appears to be a preference for dimensionality reduction techniques in the broader landscape. [58, 59]. These techniques, including Uniform Manifold Approximation and Projection for Dimension Reduction, t-distributed stochastic neighbour embedding (tSNE/ viSNE), are dimensionality reduction algorithms [60], whereas FlowSOM (Self-Organizing Map) and Spanning-tree Progression Analysis of Density-normalized Events (SPADE) cluster cells based on similarities in their surface markers [61–65]. They have become a prominent feature in the analysis of single-cell technologies, including flow and mass cytometry, as well as scRNA-seq (Figure 4). They are often considered an improved alternative to manual gating, as they offer an unbiased and systematic exploration of the data [64]. For instance, Dzangué-Tchoupou and colleagues [58] performed comprehensive immune profiling of peripheral blood cells from 18 IBM patients, 26 other IIM patients and 16 HC through mass cytometry. By leveraging SPADE, CITRUS and classification and regression trees (CART) algorithms, along with receiver operating characteristics curves, they identified that a frequency of CD8^+^, T-bet^+^ cells exceeding 51.5% provided a potential diagnostic biomarker specific to IBM exhibiting high sensitivity and specificity. Similarly, Wilfong *et al.* [59], dissected mass cytometry data by integrating t-SNE, CITRUS and marker enrichment modelling (MEM). The authors revealed shared immunological features across 17 IIM patients (6 DM, 4 PM, 7 ASS) including a decreased expression of the activation marker CD180 on B cells and the homing marker CXCR3 on T cells, relative to healthy controls. Additionally, two distinct subgroups of IIM patients could be delineated. The first group demonstrated an upregulation of CXCR4 across all cell populations, with the authors suggesting this upregulation may be associated with increased diseased severity. Alternatively, increased frequency of the CD19^+^, CD21^lo^, CD11c^+^ and CD3^+^, CD4^+^, PD1^+^ delineated the second IIM subsets and represented a pro-fibrotic phenotype [59].

Supervised classification algorithms, like SVMs and random forests (RF), are also popular methods for analyzing immunophenotyping data. These algorithms recognize patterns and complex relationships between surface and intracellular marker expression, fuelling predictive models for disease diagnosis and prognosis. In a study by Ye and colleagues, immune signatures were scrutinized in 82 amyopathic dermatomyositis with interstitial lung disease (ADM-ILD) patients and 82 HC [66]. Patients were stratified based on their immune cell subset frequencies using hierarchical clustering analysis followed by supervised ML methods (Balanced Random Forest Model) to identify the subsets of predictive value. The study identified two distinct clusters correlating with different disease activities and clinical outcomes in ADM-ILD. Cluster 1 was characterized by an enrichment of activated CD45RA^+^, HLA-DR^+^ and CD8^+^ T cells with decreased proportion of the CD56^dim^ NK cell subset that correlated with a higher prevalence of rapidly progressive ILD and higher mortality rate. In contrast, cluster 2 was characterized by abundant non-activated T cells and had favourable clinical outcomes with survival rate over 6 years higher than cluster 1. These findings suggest that peripheral immunological features may be used to stratify ADM-ILD patients and correlate with differential disease severity and clinical outcomes [66]. Through hierarchical clustering and Balanced Random Forest Models, distinct clusters surfaced, bearing correlations with different disease activities and outcomes. Notably, these clusters showcased variety of immune cell subset frequencies, each tied to divergent prognosis. Similar strategies were adopted in delineating 421 DM patients with anti-MDA5 antibodies, into three distinct prognostic clusters based on lymphocyte counts [67]. Specifically, the arthritis-associated cluster demonstrates elevated lymphocyte counts and boasts the most favourable prognosis, suggesting a subset with a more positive disease trajectory. In contrast, the rapidly progressive interstitial lung disease (RP-ILD) cluster is characterized by the lowest peripheral lymphocyte levels and an unfavourable prognosis, highlighting a subgroup with heightened disease severity. Additionally, the cluster associated with the typical DM rash presents a moderate peripheral lymphocyte count, indicating an intermediate prognosis—offering a nuanced understanding of disease outcomes within this particular subgroup.

Overall, immunophenotyping has become an essential tool for identifying novel biomarkers and understanding the complex immune system dysregulation that occurs in inflammatory myopathies. Dimensionality reduction and ML algorithms, particularly those involving unsupervised clustering, have revolutionized the way researchers approach data analysis in single-cell technologies. They allow for the unbiased identification of cell populations, which provides insights into the aetiopathology of disease, offering new avenues for the more accurate diagnosis and identification of novel therapeutic targets. Moreover, these findings underscore the heterogeneity of inflammatory myopathies and the potential utility of distinct biomarker profiles in predicting and managing diverse clinical trajectories.

### Leveraging the power of ML on multi-omic data helps unveil mechanism-based pathways in IIM

Multi-omics profiling studies is a rapidly emerging field that aims to integrate data from genomics, transcriptomics, proteomics and metabolomics to obtain a comprehensive understanding of biological systems. The generation of multi-omic meta datasets has significantly increased the complexity of analysis, which demands greater computational power for processing and analysis. The majority of multi-omic studies in IIM have utilized supervised classification-based methods such as SVMs, linear regression and RFs as well as dimensionality reduction methods such as Partial Least Squares Discriminant Analysis (PLS-DA). These methods identify patterns and complex relationships in the data that would be difficult to identify using traditional statistical methods alone.

Using high-throughput RNA sequencing in muscles isolated from 18 IMNM patients and 10 HC, Chen and coworkers [68] identified 193 differentially expressed genes associated with inflammatory immune responses, cardiac muscle contraction, skeletal muscle regulation and lipoprotein metabolism. Three feature genes, *LTK, MYBPH* and *MYL4* that are associated with the autophagy-lysosome pathway and muscle inflammation were identified as potential biomarker genes for IMNM with an accuracy of 97.3% using the least absolute shrinkage selection operator (LASSO) and SVM-recursive feature elimination (SVM-RFE) algorithms [68].

Pinal-Fernandez and colleagues applied ML algorithms to muscle isolated from 20 HC and 119 myositis patients (39 with DM, 49 with IMNM, 18 with anti-Jo1-positive AS and 13 with IBM). RNA-transcriptomic analysis found over 10,000 unique gene expression patterns that distinguish DM, AS, IMNM and IBM from HC [69]. The support vector ML algorithm demonstrated >90% accuracy in classifying patients. Further investigations using recursive feature elimination identified genes that were overexpressed in one type of myositis. For instance, CAMK1G, EGR4 and CXCL8 transcripts were increased in AS, but neither in DM nor in other types of myositis. Additionally, the same method identified genes uniquely overexpressed in various MSA-defined myositis subtypes, such as APOA4 was found to be significantly overexpressed in anti-HMGCR positive myopathy, and mucosal vascular address in cell adhesion molecule 1 (MADCAM1) was found overexpressed in anti-Mi2 positive DM. These findings demonstrated the potential of ML to identify genes related to specific myositis types and MSA-defined subtypes [69].

In addition to genomics, other investigative approaches include metabolomics. It can be applied to various biofluids, including blood and urine that are more easily accessible compared to invasive muscle biopsies and traditional histological analysis. ML models have emerged as valuable tools for identifying biomarkers and unravelling molecular mechanisms from metabolomic data. In a recent study conducted by Liu et al., supervised classification algorithms such as RF and AdaBoost were effectively employed to detect perturbations in metabolic pathways across various subtypes of IIMs. The study encompassed 52 healthy donors and 79 major IIM subtypes, including DM, ASS, IMNM and MSA-defined subtypes, such as anti-Mi2^+^, anti-MDA5^+^, anti-TIF1γ^+^, anti-Jo1^+^, anti-PL7^+^, anti-PL12^+^, anti-EJ^+^ and anti-SRP^+^. The analysis revealed significant disturbances in fatty acid biosynthesis in both plasma and urine samples, with several metabolites exhibiting differential expression across various IIM subtypes. Notably, creatine in plasma was identified as a potential specific biomarker for the INMN while tiglylcarnitine in urine showed promise as a distinctive biomarker for anti-glycyl tRNA synthetase (anti-Ej) subtype of ASS. Additionally, 16 shared metabolites were detected among the plasma and urine samples of different IIM subtypes [70].

Kang et al. [71] conducted a comparative study using ML techniques to identify metabolic differences among IIM patients, 30 ankylosing spondylitis (AS) patients and 10 HC. They employed supervised ML models, including linear regression, RF and SVMs, and discovered seven distinct metabolites, including branched-chain amino acids (BCAAs), biogenic amines and lipids, that effectively distinguished IIM patients from both healthy controls and AS groups. Notably, elevated levels of specific amino acids, like BCAAs, were associated with inflammation through mTORC1 activation. The study also explored metabolic changes in skeletal muscles using a mouse model of IIM induced by C-protein immunogens, identifying 68 significantly altered metabolites. Pathway analysis indicated a significant decrease in spermine and spermidine levels, indicative of polyamine pathway down-regulation. Furthermore, changes in metabolites related to beta-alanine and histidine metabolisms suggested potential muscle cell damage during inflammation [71].

In another study, a combined metabolomic and transcriptomic analysis of 14 IBM muscle samples revealed specific metabolic alterations. [72]. Employing the widely used Partial Least Squares Discriminant Analysis (PLS-DA) model, the researchers deciphered complex relationships, identifying 198 metabolites linked to upregulated histamine biosynthesis and were associated with an accumulation of mast cells in IBM. The glycosaminoglycan pathways were notably upregulated, as evident from the excessive chondroitin sulphate levels observed in both metabolomic and transcriptomic analyses. Histopathological examinations further corroborated these findings, confirming the presence of substantial chondroitin sulphate accumulations within the muscle tissues of IBM patients. Notably, deficiencies in key energy metabolism molecules, carnitine and creatine, were also unveiled, suggesting potential biomarker avenues for IBM treatment through diet supplementation [72].

The field of multi-omics profiling has rapidly evolved to gain a comprehensive understanding of IIM by integrating data from genomics, transcriptomics, proteomics and metabolomics. Noteworthy findings include identification of genetic biomarkers associated with the autophagy–lysosome pathway and muscle inflammation in IMNM. Pinal-Fernandez and colleagues utilized ML algorithms to classify distinct myositis subtypes based on unique gene expression patterns [69]. Metabolomic studies revealed perturbations in metabolic pathways across IIM subtypes, with specific biomarkers identified for IMNM and different MSA-defined IIM subtypes [69, 70]. The combined metabolomic and transcriptomic analysis in IBM uncovered specific metabolic alterations associated with histamine biosynthesis, glycosaminoglycan pathways and deficiencies in key energy metabolism molecules presenting potential therapeutic avenues. These studies collectively highlight the power of multi-omics approaches and ML techniques in uncovering intricate molecular signatures, biomarkers and potential therapeutic targets.

### ML approaches for analyzing medical images in IIMs

**Figure 5:**
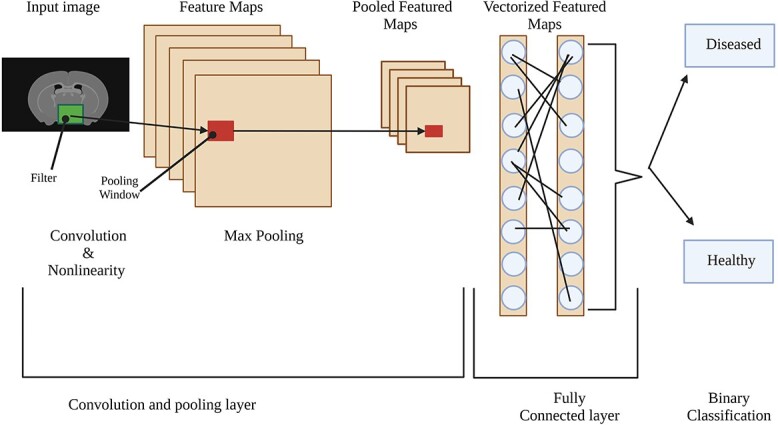
Building blocks of typical CNN from an image. Convolutional layer: (**A**) set of filters are learned during training and applied to the input image to extract features at different spatial locations. Each filter convolves over the input image to produce a feature map. Pooling layer: The pooling layer is used to down-sample the output of the convolutional layer, reducing the spatial dimensions of the feature maps while retaining the important features. Fully connected layer: The fully connected layer is used to produce the final output of the network. It takes the flattened output from the previous layer and applies a set of weights to produce a vector of outputs.Figure created with Biorender.com

ML has been widely used to analyze medical images for tasks such as segmentation, classification and diagnosis. Deep learning models, particularly convolutional neural networks (CNN), have shown great success in various medical imaging applications, including radiology, ophthalmology and pathology [73]. CNNs are specifically designed to work with images, and their success lies in their ability to learn hierarchical representations of visual features directly from the raw input data (Figure 5). They have shown superior performance compared to traditional ML methods such as SVMs and RFs. Kabeya and colleagues trained a CNN on muscle biopsy images from patients with PM, DM and IBM, as well as healthy controls. This model accurately differentiated these IIMs from hereditary muscle diseases, with a sensitivity and specificity that outcompeted specialist physicians [74].

Texture image analysis (TA) is a common method used in radiomics, which involves the extraction of quantitative features from digital imaging data (CT, MRI, ultrasound, PET) to characterize the underlying tissue properties [75]. TA quantifies texture attributes like roughness or smoothness, furnishing supplementary diagnostic or prognostic insights. Nagawa et al. [76] employed TA on MRI data from 55 IIM and 19 non-IIM patients. Several ML models classified TA features, unveiling disease activity trends in IIM subgroups and distinguishing anti-Jo-1 and anti-aminoacyl tRNA synthetases (ARS) IIM subgroups. However, it showed limited ability to differentiate IIM from non-IIM samples.

Deep learning, like unsupervised novelty detection (ND), aids medical image analysis by training on healthy data to detect deviations. For example, Burlina and colleagues used ND on 3586 ultrasound images obtained from 89 subjects, including 35 controls and 54 with myositis, achieving a baseline (ROC AUC of 71.92% and 95% CI error margin). These promising results indicated the potential of implementing this method as a prescreening tool for myopathies [77]. In a similar study, a DL neural network applied to whole-body MRI achieved correct classification percentages of 69–77%, and comparable diagnostic prowess to radiologists in distinguishing facioscapulohumeral muscular dystrophy (FSHD1) from myositis. DL even corrected radiologists’ misclassifications, showcasing its efficacy to generate accurate diagnosis from MRI data [78].

In conclusion, ML has shown its potential to become an indispensable tool in medical image analysis, providing significant advantages over classical human-made analysis of IIM biopsies. DL models such as CNNs have proven to be highly effective in distinguishing between different types of muscle diseases, and in some instances outcompeted specialist physicians. TA analysis provides additional information to aid in diagnosis or prognosis by quantifying the underlying tissue properties. Unsupervised ND provides a promising prescreening tool for myopathies given its effectiveness at identifying abnormal or novel patterns in imaging data. These advanced ML techniques applied to imaging have the potential to, providing faster and more accurate diagnoses and prevent patient discomfort associated with invasive conventional muscle biopsy.

### Machine learning models for predicting patients’ response to treatments

Beyond biomarker discovery for disease diagnosis and prognosis, biomarkers can assist clinicians to make informed decisions regarding patient treatment strategies. ML has also proven useful in predicting treatment responses. For instance, in a study of 51 IIM (DM, PM, ASS, INMN) patients. Demographic, clinical and serological parameters were evaluated to determine the most effective predictors of patients' response to intravenous and subcutaneous administration of immunoglobulins [79]. Previously, the evaluation of five supervised ML models showed that elastic net regression, which combines features of both Ridge regression and Lasso regression, was the most effective model for this application [80]. The authors determined that dysphagia, skin disorders and the myositis activity index (MITAX) were good predictors of muscle strength (as measured by the manual muscle testing of eight groups (MMT8)) and found that IVIg therapy yielded better results in patients with more active systemic disease [79].

Anti-SRP antibody-positive IMNM patients are refractory to corticosteroids [43], and several clinical risk factors are identified with refractory disease including, being male, severe muscle weakness and concurrent ILD. In addition, the extent of fatty infiltration of thigh muscles over time have been identified as predictors of treatment response. ML algorithms have been used to analyze these pathological factors. Elevated expression of B cell activating factor receptor (BAFF-R) in muscle tissue has been identified as predictors of refractory SRP-positive IMNM patients. Leveraging these refractory related factors and using ML-based predictive models may critically help healthcare professionals to better identify risk-patients and adjust care plans.

### ML approaches for predicting comorbidities

IIM’s are complex multisystem autoimmune disorders, involving inflammation and immune system dysfunctions that mainly not only impact skeletal muscle but also affect other tissues and organs, including skin, joints and lungs [81]. Given the spectrum of systems that can be affected, individuals with IIM often experience comorbidities. These comorbidities can range from rheumatic diseases to ILD, reinforcing the multifaceted nature of IIM. Understanding and managing these comorbidities are essential aspects of comprehensive patient care.

As previously mentioned, certain subtypes of myositis are associated with an increased risk of malignancy, such as DM patients with anti-TIF1-γ [46]. It is estimated that one third of myositis patients will develop a malignancy. In fact, malignancy is the leading cause of death in adults with IIM [81]. Zhao et al. employed various ML techniques, including Sankey diagrams, elastic net, RF, multidimensional scaling and hierarchical clustering, to categorize subtypes of anti-TIF1-γ^+^ myositis and assess the most critical factors for predicting cancer risk [46]. Among the patients studied, 54% had cancer, typically diagnosed within 6 months of myositis diagnosis. The anti-TIF-1γ^+^ myositis patients were grouped into low, intermediate, or high cancer risk subtypes. Key ML classifiers included disease duration, blood lymphocyte percentage, neutrophil percentage, neutrophil-to-lymphocyte ratio, gender, C-reactive protein (CRP) levels, shawl sign, arthritis/arthralgia, V-neck sign and anti-PM-Scl75 antibodies. Notably, RF achieved an accuracy of over 90%, underscoring the potential of ML models in aiding physicians in selecting appropriate cancer screening strategies for anti-TIF-1γ^+^ myositis patients [46].

Also, Zhang and colleagues performed LR modelling in a cohort of 168 IIM patients including DM, PM, ASS and IMNM to determine the key features that could be used for malignancy prediction [82]. Three predictors (age, alanine aminotransferase (ALT) < 80 U/L and seropositivity for anti-TIF-1-γ antibodies) were identified as positive predictors for malignancy while, ILD was found to be a negative predictor of malignancy. The LR model was as good or better than the other ML models including RF, neural network and extreme gradient boosting at predicting malignancy. The AUC of the ROC was determined at 78.4% [82].

In another study, the researchers examined the medical records of 397 patients with IIM to identify potential risk factors for ILD, other rheumatic diseases and malignancies [83]. Antibodies such as anti-PM/Scl, anti-Ro52, anti-aminoacyl-tRNA synthetase and anti-MDA5 constituted risks for ILD. Patients with Raynaud's phenomenon, arthralgia and anti-nuclear antibodies were found to be prominent risk factors for other overlapping rheumatic diseases. For IIM patients with associated malignancies, being male and the presence of anti-TIF-1-γ antibodies were risk factors. Hierarchical clustering generated a subclassification into six subgroups including (1) malignancy overlapping DM, (2) classical DM, (3) PM with severe muscle involvement, (4) DM with ILD, (5) PM with ILD and (6) overlapping of myositis with other rheumatic diseases [83].

Overall, biomarkers can help serve as ML modelling provides numerous benefits to healthcare providers, such as rapidly identifying patients who stand to gain from specific treatments, or alternatively may be susceptible to adverse reactions. Additionally, ML models can help discern patients at risk of certain comorbidities, enabling implementation of targeted interventions.

### Advantages and limitations of ML for biomarker discovery

As ML algorithms become increasingly prevalent in the biomedical field, it is important to note that the implementation and interpretation of these models requires both expertise in data analysis and domain-specific knowledge. Additionally, the accuracy and generalisability of these models rely heavily on the quality and quantity of data available for training and testing [9]. For instance, in transcriptomics studies, reference genomes may lack complete annotations for certain genes or regions, leading to the complete omission of important transcripts. Furthermore, quantification challenges, such as accurate measurement of low-abundance transcripts and susceptibility to noise, further compound these issues. Additionally, data acquisition method transparency with detailed methodology description is essential to enable methods generalization, and data reproducibility is essential, as variations in sequencing platforms and bioinformatics pipelines can introduce biases. While the potential benefits of using ML for biomarker discovery are numerous, it is important to carefully consider the limitations and potential biases inherent in these models (Table 2).

**Table 2:** Advantages and limitations of ML for biomarker discovery

Advantages	Limitations
Can handle large amounts of data	May require significant computational resources
Can detect complex patterns in data	May be prone to overfitting or underfitting data
Can be used for real-time prediction	May require significant training time
Can improve diagnostic accuracy	May be limited by the quality and completeness of data
Can identify new biomarkers and disease subtypes	May require expertise in data analysis and machine learning
Can tailor treatment plans for individual patients	May not be able to capture all relevant variables in the data
Can reduce human error and bias	May raise ethical concerns about the use of AI in healthcare
Can be applied to diverse types of data (e.g. imaging, genomics)	May be limited by the availability of high-quality data
Can accelerate drug discovery and development	May require collaboration between researchers with different expertise

Furthermore, the lack of standardization in ML modelling for biomarker discovery is a significant challenge. There is often variability in the selection of features, model training and evaluation metrics, leading to inconsistent or conflicting results. Moreover, different ML algorithms may perform differently depending on the dataset and the specific research question, making it difficult to identify the best approach. Efforts are being made to address these issues, including the development of standardized protocols for data sharing and analysis, and the establishment of benchmark datasets for evaluating the performance of ML algorithms [84].

Overall, using ML algorithms to assist and complement conventional human interpretation can help to improve the accuracy, efficiency of biomarker discovery, and may lead to new insights into disease mechanisms and potential therapeutic targets for IIM patients. While ML algorithms have the potential to revolutionize biomarker discovery, it is important to carefully consider the caveats, limitations and ethics of using these algorithms and to validate the results with conventional human interpretation.

## CONCLUSION

In conclusion, the integration of ML in biomarker discovery for IIMs holds tremendous promise for advancing our understanding of these complex diseases. ML techniques have demonstrated efficacy in predicting features that can be incorporated into innovative diagnostic criteria and evaluating the specificity and sensitivity of these criteria. Numerous studies have underscored the clinical significance of MSAs as diagnostic, prognostic and predictive biomarkers, enabling clinicians to tailor treatment plans and address patient comorbidities effectively. Furthermore, advancements in medical image analysis present non-invasive alternatives for diagnosing IIM rapidly and effectively. Overcoming the challenges posed by the heterogeneity among patients, ML-based predictive modelling, driven by high-dimensional data from immunophenotyping and multi-omic studies, has unveiled novel biomarkers.

However, the diverse landscape of experimentation and testing, particularly in autoantibody detection and RNA-based transcriptomic approaches, calls for the establishment of standardized protocols as imperative to ensure the reproducibility and comparability of results across studies. This is especially crucial for the robust implementation of ML-based predictive modelling, which relies heavily on consistent and high-quality data inputs. Addressing these standardization challenges is essential for fostering collaboration among researchers and clinicians, facilitating the pooling of data and ultimately enhancing the reliability of biomarker discoveries.

Looking forward, the establishment of patient registries becomes crucial for comprehensive data collation, and the integration of AI/ML into these registries can provide direct feedback to clinicians, contributing to personalized treatment strategies. While challenges and limitations persist, the ongoing application of ML in IIM research has the potential to revolutionize our understanding of these diseases, paving the way for more targeted and efficacious therapies, provided that standardized protocols are implemented and adhered to across the scientific community.

**Key Points**

- Integrating of ML into biomarker discovery for IIMs holds great potential for refining current diagnostic paradigms, predicting prognosis and tailoring targeted and effective treatment strategies.
- ML-driven predictive modelling with high-dimensional data reveals novel biomarkers, providing nuanced insights into the diverse IIM patient population and overcoming challenges presented by its heterogeneity.
- By leveraging ML, medical image analysis offers rapid and effective non-invasive alternatives for diagnosing.
- Careful consideration must address the limitations and biases inherent to ML models, emphasizing robust validation strategies, transparent documentation of data sources and continuous refinement to ensure reliable outcomes.
- Implementing standardized protocols across all data, especially in autoantibody detection and transcriptomics, is essential. This standardization plays a critical role to ensuring the reproducibility of ML-driven predictive modelling, thereby bolstering the overall reliability of biomarker discovery in IIMs.

## Data Availability

The machine learning sunburst plot presented in this article is *based on the data available in the GitHub repository hosted by EmilyJane994*. The repository, titled “Machine-learning-sunburst-plot,” can be accessed at the following URL: https://github.com/Emilyjane994/Machine-Learning-sunburst-plot DOI: 10.5281/zenodo.10445877. Additionally, *datasets that were derived from sources in the public domain (Pubmed) can be found here:*  https://pubmed.ncbi.nlm.nih.gov/?term=Machine+Learning+%26+Inflammatory+myopathies.
