# Correction to: From data to diagnosis: how machine learning is revolutionizing biomarker discovery in idiopathic inflammatory myopathies

**DOI:** 10.1093/bib/bbae100

**Published:** 2024-03-05

This is a correction to:

Emily McLeish, Nataliya Slater, Frank L Mastaglia, Merrilee Needham, Jerome D Coudert, From data to diagnosis: how machine learning is revolutionizing biomarker discovery in idiopathic inflammatory myopathies, *Briefings in Bioinformatics*, Volume 25, Issue 1, January 2024, bbad514, https://doi.org/10.1093/bib/bbad514

In the originally published version of this manuscript, The affiliations of author, Jerome D. Coudert, should be corrected as follows:

a. Murdoch University, Centre for Molecular Medicine and Innovative Therapeutics, Murdoch, Western Australia (WA), Australia

b. Perron Institute for Neurological and Translational Science, Nedlands, WA, Australia

c. University of Notre Dame Australia, School of Medicine, Fremantle, WA, Australia

This error has been corrected.

